# Supplementary material for: GPER Agonist G1 Prevents Wnt-Induced JUN Upregulation in HT29 Colorectal Cancer Cells
Source: Int J Mol Sci. 2022 Oct 20;23(20):12581. doi: 10.3390/ijms232012581 (PMC9603962; doi:10.3390/ijms232012581)
Supplement: Supplementary file 1 [file ijms-23-12581-s001.zip › ijms-1910410-supplementary.pdf]

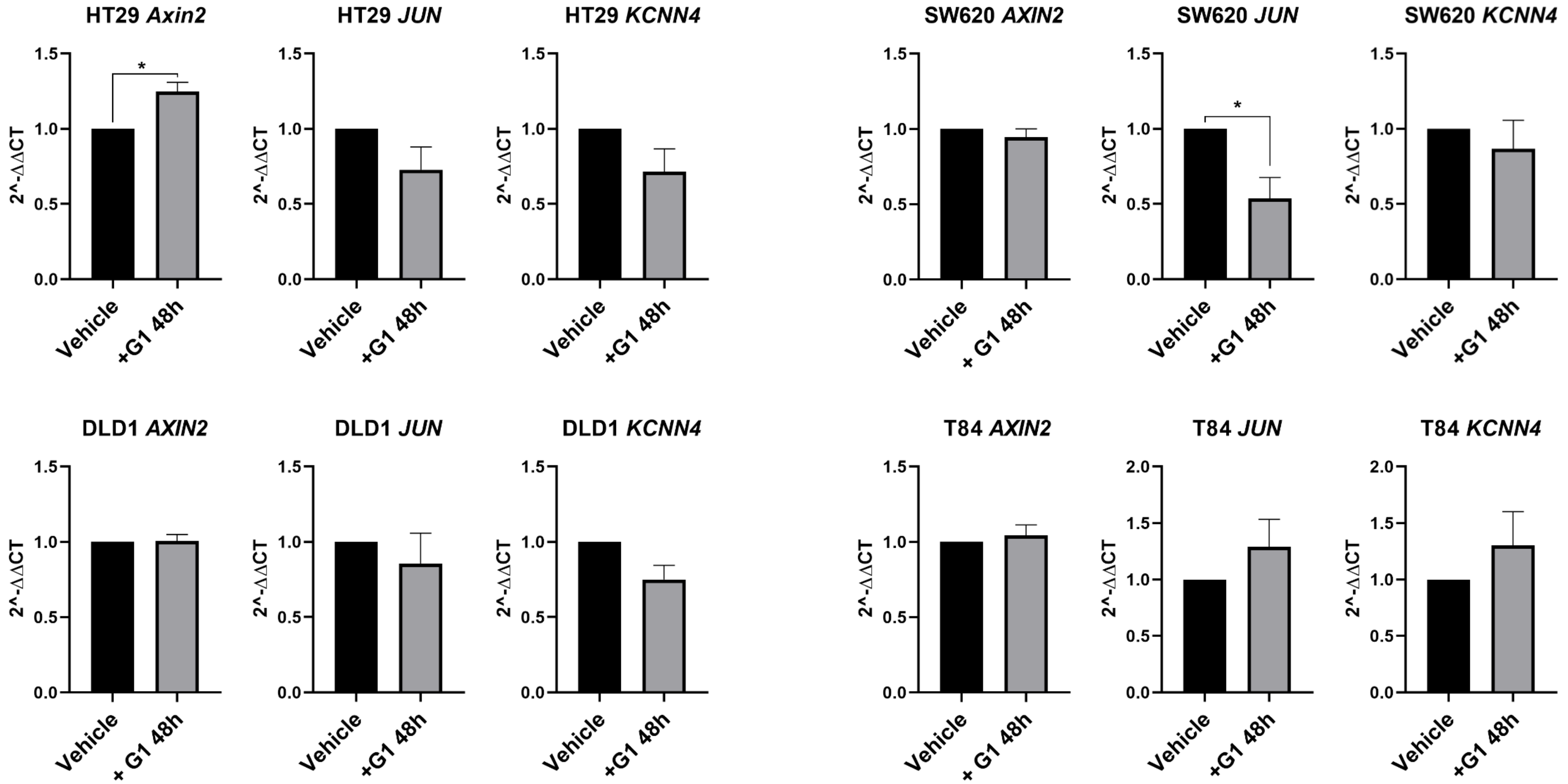

Supplementary Figure S1: Wnt target gene expression in response to G1 treatment in multiple CRC cell lines
HT29, SW620, DLD1 and T84 CRC cell lines were treated with 0.25μM G1 or Vehicle (DMSO) for 48h. RT-qPCR analysis shows that in HT29 cells G1 induced an increase in *AXIN2* and a small but not statistically significant decrease in *JUN* and *KCNN4* expression. In SW620 cells, G1 treatment also induced a decrease in *JUN* expression. No other statistically significant expression changes were detected in these cell lines under the conditions tested. Statistical analysis was by student t-test with $n = 4$ for HT29, SW620 and DLD1 cells with each treatment and $n = 3$ for T84 cells. Mean ± SEM. $*p < 0.05$.
